# Gut Permeability and Immune-Mediated Inflammation in Heart Failure

**DOI:** 10.3390/biomedicines12061217

**Published:** 2024-05-30

**Authors:** Maria Perticone, Simona Gigliotti, Ermal Shehaj, Raffaele Maio, Edoardo Suraci, Sofia Miceli, Francesco Andreozzi, Giovanni Matera, Francesco Perticone

**Affiliations:** 1 Department of Medical and Surgical Sciences, University Magna Graecia, 88100 Catanzaro, Italy; andreozzif@unicz.it (F.A.); perticone@unicz.it (F.P.); 2 Department of Health Sciences, University Magna Graecia, 88100 Catanzaro, Italy; simona_gigliotti@yahoo.it (S.G.); mmatera@unicz.it (G.M.); 3 Cardiology and Cardiovascular Intensive Care Unit, Presidio Ospedaliero “Giovanni Paolo II” di Lamezia Terme, Azienda Sanitaria Provinciale di Catanzaro, 88046 Lamezia Terme, Italy; shehajermal@gmail.com; 4 Geriatrics Unit, P.O. Germaneto, Azienda Ospedaliero-Universitaria “Renato Dulbecco”, 88100 Catanzaro, Italy; raf_maio@yahoo.it (R.M.); sofy.miceli@libero.it (S.M.); 5 Internal Medicine Unit, P.O. Pugliese-Ciaccio, Azienda Ospedaliero-Universitaria “Renato Dulbecco”, 88100 Catanzaro, Italy; edoardosuraci88@gmail.com

**Keywords:** heart failure, inflammation, gut dysbiosis, Toll-like receptors

## Abstract

Heart failure (HF) is characterized by low-grade immune-mediated inflammation due to increased Toll-like receptor (TLR) expression as response to endotoxin increase and dysregulated gut barrier permeability. We investigated TLR expression and possible gut dysbiosis in HF patients compared to a control group. We enrolled 80 Caucasian HF patients and 20 controls. Low-grade immune-mediated inflammation was evaluated by TLR expression, while gut dysbiosis by the detection of zonulin and bacterial endotoxin activity in a semi-quantitative (endotoxin activity assay [EAA]) and quantitative (limulus amebocyte lysate [LAL] test) way. Compared to controls, patients with HF showed significantly higher age and blood pressure values, worse metabolic profile and kidney function, higher inflammatory biomarkers levels, and lower levels of zonulin and endotoxin activity. When dividing failing patients in those with reduced ejection fraction (HF-rEF) and those with preserved ejection fraction (HF-pEF), HF-rEF patients showed significantly higher values of inflammatory biomarkers and TLR expression than HF-pEF patients. Gut permeability biomarkers inversely correlated with the severity of HF and positively with renal function. eGFR was retained as an independent predictor of zonulin variation in all the three groups of failing patients. Present data work to extend current knowledge about the role of gut microbiota in immune-mediated inflammation in HF.

## 1. Introduction

Consistent epidemiological data have demonstrated that heart failure (HF) affects many millions of patients worldwide, causing debilitating symptoms and significantly reduced patients’ quality and expectancy of life [1]. In addition, it is very likely that the prevalence of HF will continue to raise as consequence of the increase in metabolic diseases such as obesity and type-2 diabetes mellitus, which emerge as the main risk factors for HF [2], particularly for that with preserved ejection fraction (EF). In keeping with this, in fact, the current echocardiographic HF classification defines three main forms of HF, including HF with reduced EF (HF-rEF; EF ≤ 40%), HF with preserved EF (HF-pEF; EF ≥ 50%), and an intermediate form with an EF ranging from 41 to 49% [1]. Hypertension, female sex, type-2 diabetes mellitus and obesity are major risk factors for HF-pEF, while ischemic cardiac disease is more associated with HF-rEF [3]. On the other hand, it is clinically relevant to remark that both the incidence and mortality of HF continue to increase despite preventive strategies and the availability of highly effective treatments [4]. All this implies a considerable use of economic resources, which entails a significant commitment for all national health systems [5].

In recent decades, it has been well established that HF patients, independently from prevalent systolic or diastolic dysfunction, are characterized by a low-grade chronic inflammation that contributes to both the maintenance and progression of HF [6,7]. This inflammatory status, probably driven by the coexistence of traditional cardiovascular (CV) risk factors, is maintained by other mechanisms, such as immune system activation [7,8,9]. In accordance with this, our group recently demonstrated a higher activation and expression of Toll-like receptors (TLRs), in particular TLR2 and TLR4, in HF patients compared to a control group, and these differences were more evident in patients with HF-rEF [10]; this overexpression of TLRs induces, in turn, the gene transcription of pro-inflammatory cytokines [11], following the recognition of pathogen-associated molecular patterns (PAMPs) [8,9]. Specific PAMPs recognized by members of the TLRs family have been widely characterized, and several data points demonstrate that TLR4 mediates the response to lipopolysaccharide (LPS) [12], a glycolipid constituent of the Gram-negative bacteria wall cells [13]. In addition, it is well demonstrated that the increase in circulating LPS levels may be considered as an expression of the disruption of normal intestinal permeability with consequent elevated translocation of both bacteria and bacterial toxins that, stimulating the immune system via the activation of nuclear factor-κb (NF-κb), promote systemic inflammation [14,15,16]. In addition, it is important to remark that increased LPS levels are a consequence of a parallel increase in zonulin levels, a protein released by epithelial cells of the small intestine involved in the increased gut permeability, particularly expressed in all chronic conditions characterized by an inflammatory status [17,18]. Thus, serum zonulin levels can be considered as an indirect measure of gut permeability. Moreover, several data points demonstrate that HF is associated with alterations of gut microbiota and consequent alterations of gut barrier permeability, so-called gut dysbiosis [19,20].

In accordance with these findings, the involvement of LPS in cardiovascular [21] and metabolic diseases [22,23] has been widely demonstrated, but, to our knowledge, at this moment, a clear cause–effect relationship between gut dysbiosis and HF progression has never been documented. Thus, we designed the present study to investigate whether the increased immune-mediated inflammation (evaluated through TLRs activity) observed in HF patients may be considered as a response to gut dysbiosis, evaluated by zonulin and indirect LPS levels, and whether there are differences in these two parameters between HF patients (both those with HF-rEF and HF-pEF) in comparison with a control group.

## 2. Materials and Methods

In this study, we enrolled 80 consecutive Caucasian HF outpatients, 47 males and 33 females with a mean age of 64.5 ± 5.3 years, and 20 healthy subjects, 13 males and 7 females, aged 54.8 ± 5.9 years, referred to our University Hospital for the evaluation of their cardiometabolic risk profile. 

Exclusion criteria were history of autoimmune diseases and all conditions associated with immune system activation, cardiac storage diseases, congenital cardiomyopathies, malignancies, thyroid dysfunctions, liver insufficiency, chronic inflammatory bowel diseases, and abuse of alcohol and drugs. 

Diagnosis of HF was carried out according to the recent guidelines of the European Society of Cardiology, considering the presence of symptoms and/or signs, as well as echocardiographic parameters. LPS activity was tested by two different methods, endotoxin activity assay (EAA) and limulus amebocyte lysate (LAL), in order to obtain a semi-quantitative and a quantitative measure, respectively. The Ethical Committee approved the protocol, and informed written consent was obtained from all participants. All the investigations were performed in accordance with the principles of the Declaration of Helsinki. 

### 2.1. Echocardiographic Measurements

All echocardiographic readings were obtained using a VIVID-7 Pro ultrasound machine (GE Technologies, Milwaukee, WI, USA), with patients in a partial left decubitus position, by an experienced sonographer (SM) not aware of patients’ clinical data, to optimize the measurements’ reproducibility. Tracings were taken in a random order with an annular phased array 2.5 MHz transducer and, for this analysis, were considered only frames with optimal visualization of cardiac structures. For each parameter of every patient, we computed at least five measurements. Readings were obtained under 2-dimensional guidance, and M-mode measurements were taken at the tip of the mitral valve or just below. Evaluation of interventricular septum (IVS) thickness, posterior wall (PW) thickness, and left ventricular internal diameter (LVID) were made at end-diastole and end-systole. Left ventricular mass (LVM) was calculated using the Devereux formula [24] and normalized by body surface area (i-LVM). Measurements of EF were obtained by Simpson method. 

### 2.2. Laboratory Determinations

All laboratory determinations were obtained after at least 12 h of fasting. The glucose oxidation method (glucose analyzer, Beckman Coulter, Milan) was used to determine glucose values; while an enzymatic method (Roche Diagnostics GmbH, Mannheim, Germany) was used to evaluate total, low-density lipoprotein (LDL), and high-density lipoprotein (HDL) cholesterol and triglyceride concentrations. Creatinine and uric acid were measured using the Jaffe methodology. Estimated glomerular filtration rate (e-GFR) values were obtained by the CKD-EPI equation. High-sensitivity C-reactive protein (hs-CRP) levels were measured with an automated instrument (Cardio- Phase hs-CRP, Siemens Healthcare, Erlangen, Germany). N-terminal brain natriuretic peptide (NT-proBNP) levels were evaluated by double-antibody sandwich ELISA method. Fibrinogen was measured with a coagulation method on the instrument BCSXP. The complete blood count was performed by flux cytometry. 

### 2.3. Serum Zonulin Assay

Human zonulin ELISA kit (Bioassay Technology Laboratory, Shangai, China) was used for the accurate quantitative detection of human zonulin. The plate was pre-coated with human zonulin antibody for 90 min at 37 °C. Then, Streptavidin-Horseradish Peroxidase (HRP) was added and bound to the biotinylated zonulin antibody. After incubation, unbound Streptavidin-HRP was washed away. Substrate solution was then added. The reaction was terminated by addition of acidic stop solution, and absorbance was measured at 450 nm. Both intra- and inter-assay coefficients of variation were <10%.

### 2.4. Endotoxin Activity Assay

The endotoxin activity assay (EAA) (SPECTRAL Medical, Toronto, Canada) is a rapid in vitro diagnostic test that uses a specific monoclonal antibody to measure the endotoxin activity in EDTA whole blood specimens. This test assesses the level of endotoxin activity in the blood specimen of a specific patient in a semi-quantitative way. The EAA uses the biological response of the neutrophils in a patient’s blood to an immunological complex of endotoxin and exogenous antibody as a measure of the endotoxin activity in the patient. The assay reacts specifically with the Lipid A moiety of LPS of Gram-negative bacteria and does not cross-react with cell wall constituents of Gram-positive bacteria and other microorganisms. A basal activity measurement (Tube 1) in the absence of the specific anti-endotoxin antibody measures the non-specific oxidative burst of the patient’s neutrophils. The test measurement (Tube 2) includes the specific antibody to measure the actual level of endotoxin activity. An additional control measurement including the specific anti-endotoxin antibody and an excess of exogenous endotoxin (Tube 3) measures the maximum oxidative burst of the patient’s neutrophils. The EAA result is calculated by normalizing the chemiluminescence in the test sample (Tube 2) against the maximum chemiluminescence (Tube 3), correcting both measurements for the basal activity chemiluminescence (Tube 1). 

### 2.5. Limulus Amebocyte Lysate (LAL) Test

To accurately detect levels of endotoxin in serum samples, we used the Thermo Scientific Pierce Chromogenic Endotoxin Quant Kit (Thermo Fisher Scientific, Waltham, MA, USA), a quantitative, efficient endpoint assay that uses amebocyte lysates derived from blood of the horseshoe crab. Amebocyte lysates are widely used as a simple and sensitive assay for the detection of endotoxin lipopolysaccharide (LPS), the membrane component of Gram-negative bacteria. When endotoxin encounters the amebocyte lysate, a series of enzymatic reactions result in the activation of Factor C, Factor B and pro-clotting enzyme. The activated enzyme catalyzes the release of p-nitroaniline (pNA) from the colorless chromogenic substrate, Ac-Ile-Glu-Ala-Arg-pNA, producing a yellow color. After stopping the reaction, the released pNA is photometrically measured at 405 nm. The developed color intensity is proportional to the amount of endotoxin present in the sample and can be calculated using a standard curve (linear regression).

### 2.6. Evaluation of TLR2 and TLR4 Expression

From fresh peripheral blood samples, within 2 h of blood collection, we isolated by Ficoll gradients, in agreement with the manufacturer’s instruction, peripheral mononuclear blood cells (PBMCs). After their separation, the PBMCs were harvested and suspended in phosphate-buffered saline (PBS) to analyze by FACS TLR expression. To identify monocytes within the isolated population, PBMCs were labeled with a phycoerythrin (PE)-conjugated anti-CD14 antibody and a fluorescein-isothiocyanate-conjugated anti-TLR2 antibody (FICT), or an allophycocycin-conjugated anti-TLR4 antibody (APC). To exclude non-specific bonds, an ISO CD14 (PE) non-specific antibody was employed as a control isotype. Samples were acquired with a FACSCalibur (BD Biosciences, Franklin Lakes, NJ, USA) and analyzed by Flow JO software [(LLC), https://www.flowjo.com/solutions/flowjo accessed on 25 April 2024]. The monocyte population was identified by both forward (FSC) and side scatter (SSC). TLR expression was considered as the ratio of mean fluorescence intensity (MFI) of the sample and MFI of the isotype control.

### 2.7. Statistical Analysis

Descriptive clinical and biological data are reported as mean ± SD for normally distributed variables, and as percent frequency for binary data. Distribution normality was assessed with Shapiro–Wilk test. Groups were compared using unpaired *t*-test when clinical and biological data were expressed as continuous variables and the χ^2^ test for categorical variables. The relationship between two linear variables was tested by a simple linear regression analysis. To test the independent relationship between zonulin and other possible covariates, we performed a multivariate regression analysis in the whole HF population and in the two groups (HF-rEF and HF-pEF) separately. Differences were assumed to be significant at *p* < 0.05. All calculations were performed with a standard statistical package (SPSS for Mac version 21.0, Chicago, IL, USA). 

## 3. Results

The study population was firstly divided into two groups based on the presence/absence of HF and, subsequently, the group of failing patients was further divided into patients with HF-rEF and patients with HF-pEF; the results of this analysis are reported in Table 1. 

**Figure 1:**
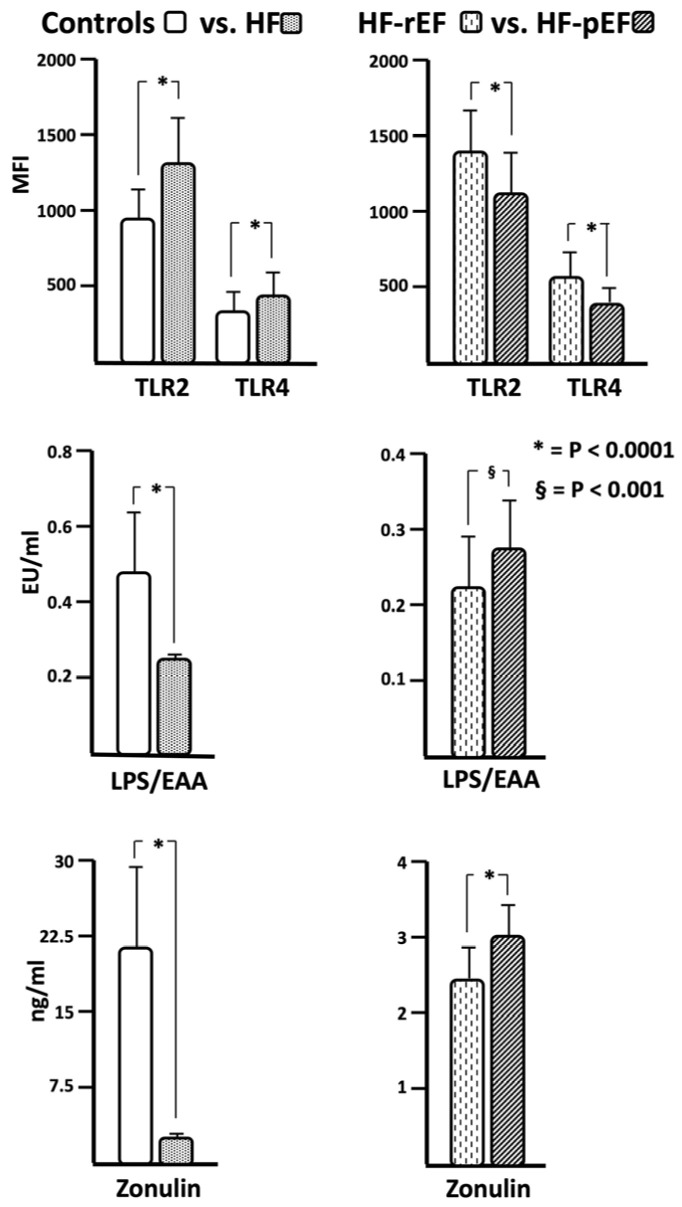
In this figure, we graphically report the mean values of Toll-like receptor 2 (TLR2) and 4 (TLT4), LPS evaluated by endotoxin activity assay (EAA), and zonulin in the four groups of the study population.

As evident, failing patients were older, with higher systolic blood pressure values (even if in the normal range), a worse metabolic profile (glucose, lipids and uric acid), and a worse kidney function compared with the control group. Similarly, patients with HF had a greater inflammatory burden, as highlighted by the significantly higher values of inflammatory biomarkers (white blood cells, high-sensitivity hs-CRP, fibrinogen) and of TLR expression. Surprisingly, HF patients showed significantly lower values of zonulin, EAA and LAL than the control group; in particular, zonulin levels were about 10-fold higher in controls subjects in comparison with the HF group; a significant difference in zonulin levels was also observed between HF-rEF and HF-pEF patients. The comparison between the two groups of HF patients showed a lower number of female subjects in the HF-rEF group, as well as lower values of both systolic and diastolic blood pressure, fasting glucose, triglyceride, and e-GFR; on the contrary, uric acid and creatinine values were higher in patients affected by HF-rEF. As expected, all inflammatory biomarkers, including TLRs, were significantly higher in the HF-rHF group than in HF-pEF one. Finally, HF-rEF patients showed significantly lower values of zonulin, EAA and LAL. In Figure 1, we graphically report the mean values of TLRs, EAA and zonulin comparing controls vs. whole HF population and HF-rEF vs. HF-pEF patients.

When considering echocardiographic parameters (Table 1), as expected, the control group showed significantly lower values of indexed LVM, end-systolic and end-diastolic LVID, IVS and PW; by definition, EF values were significantly higher in the control group. Comparison between HF groups showed that patients with HF-rEF, apart the obvious lower EF, also had lower IVS and PW thickness values, while left ventricular diameters were higher.

**Figure 2:**
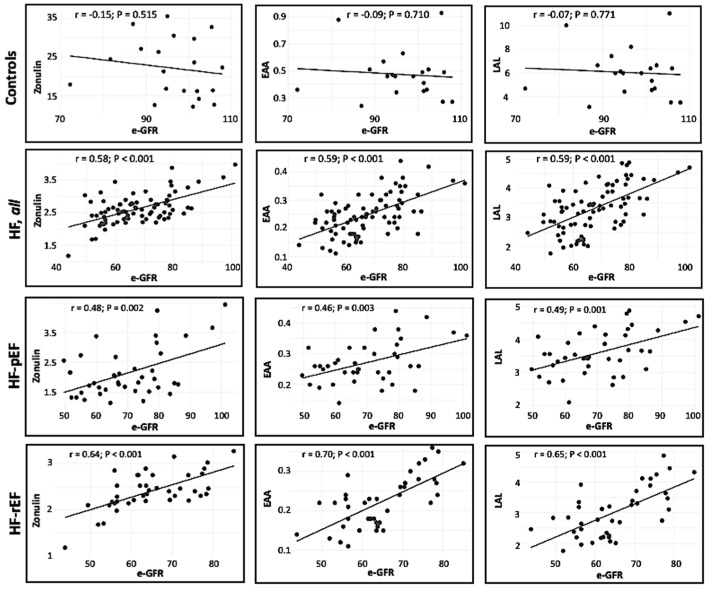
Relationship between estimated glomerular filtration rate (e-GFR) and markers of gut permeability in all the study groups.

As graphically reported in Figure 2, we observed a linear and significant relationship in the whole HF population and in the HF-rEF and HF-pEF subgroups between e-GFR, as an independent variable, and the following dependent covariates: zonulin, EAA and LAL. 

In Table 2, we report the results of correlational analysis between gut permeability markers and different covariates; interestingly, in the whole population of HF patients, but not in the subgroups of HF-rEF and HF-pEF patients, we documented a linear and significant relationship between these markers and echocardiographic ejection fraction as a possible association between HF severity and gut dysbiosis. In the same group of all HF patients, we also found a significant and linear correlation between SBP and all gut permeability markers, while a significant and inverse relationship was found between these markers and NT-proBNP, hs-CRP and fibrinogen. Interestingly, we also found a significant and linear relationship between TLRs (both TLR2 and TLR4) and EAA in the three groups of HF patients; TLR2 and TLR4 also positively correlated with LAL, but only in the two subgroups of failing patients. Thereafter, all the statistically significant covariates, with the addition of TLR2 and TLR4, were included in a multivariate regression model to test the relationship between zonulin and possible independent factors in the whole population of HF patients and in the two groups separately. Clinically relevant, eGFR was retained as an independent predictor of zonulin variation in all the three groups: β coefficient = 0.504, *p* < 0.0001 in the whole HF population; β coefficient = 0.676, *p* < 0.0001 in the HF-rEF group; β coefficient = 0.406, *p* = 0.025 in the HF-pEF group. 

**Figure 3:**
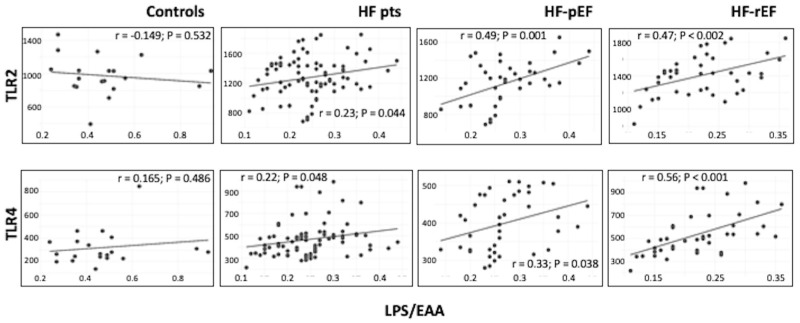
In this picture, we graphically report the linear relationship between LPS (EAA) and both TLR2 and TLR4 in the four study groups.

In Figure 3, we graphically report the linear relationship observed between LAL/EAA and both TLR2 and TLR4 in the four study groups, as confirmatory of immune system activation by PAMPs. 

## 4. Discussion

The most important finding emerging from our study is that HF patients are characterized by an increased immune-mediated inflammatory burden compared with a control group, confirming previously reported data [8,9,10]. In particular, we demonstrated that HF patients, in comparison with a control group, have a significantly higher expression of both TLR2 and TLR4; clinically relevant, this expression is significantly higher in HF-rEF patients respect to those with HF-pEF. Obviously, this different expression of TLRs is biologically plausible according with the different inflammatory burden present in the two groups of failing patients, also confirmed by the higher levels of both hs-CRP and fibrinogen found in failing patients, hypothesizing that the different cardiac morpho-functional impairment promotes different levels of low-grade immune-mediated inflammation. In this context, a crucial role in the activation of the inflammatory cytokines cascade is played by specific PAMPs, which, recognized by TLRs, activate the innate immune system as a defense response against external agents. Growing data show that TLR4 may mediate the response to LPS [12], a glycolipid constituent of Gram-negative bacterial wall cells [13].

Thus, in accordance with this, the real novelty of this study is the fact that we also investigated the activity of specific PAMPs able to activate TLRs; in particular, we considered LPS levels, with both a quantitative (LAL) and semi-quantitative (EAA) method. Surprisingly, results obtained with both methods were significantly different either when the analysis was conducted between the whole HF population and the control group, or when patients with HF-rEF and those with HF-pEF were compared, demonstrating significantly higher values of both EAA and LAL in controls vs. all HF patients, and in patients with HF-pEF when compared with HF-rEF patients, despite significantly higher expression of all inflammatory markers in the HF and HF-rEF goups, respectively. In particular, EAA and LAL levels significantly decrease with the severity of cardiac damage, showing a linear relationship with EF and an inverse relationship with NT-proBNP. Of interest, both LAL and EAA also showed a linear and significant relationship with renal function, as shown in Figure 1. These results are in agreement with those obtained in preclinical animal models, demonstrating that TLRs, an important family of pattern recognition receptors, are activated by PAMPs derived from microbial pathogens with the subsequent production of pro-inflammatory cytokines [25]. 

Several experimental and human findings have demonstrated that endotoxemia increase is attributable to gut microbiome alteration due to gut barrier permeability disruption, which, in clinical practice, is detectable by zonulin level increase [26,27]. Zonulin is a small peptide that, produced by both intestinal and hepatic cells, acts as regulator of epithelial tight junctions and its permeability with consequent bacteria translocation into the circulation. Thus, we also measured zonulin levels, which, in our study population, showed a significant progressive decrease with the severity of heart impairment and a linear relationship with renal function evaluated through e-GFR values (Figure 2) similar to that observed for EAA and LAL levels. The occurrence of all these changes of gastrointestinal function, and their associated gut microbiome alterations in failing patients, may be explained by some pathogenetic mechanisms; particularly, reduced cardiac output, increased systemic congestion and re-distribution of systemic circulation induce intestinal hypoperfusion with mucosal ischemia, which progress to increased gut permeability with subsequent augmented translocation of pathogens and bacterial toxins in the blood with the activation of immune system and inflammatory pathways. 

The inverse relationship between disrupted gut permeability biomarkers and the degree of cardiac dysfunction (Table 2) and, on the contrary, a linear relationship between these markers and renal function, evaluated using e-GFR (Figure 2) and confirmed by multivariate regression analysis that retained e-GFR, the only variable responsible for zonulin variation, are apparently different from what was expected; anyway, our data agree with those recently published by other authors in different setting of patients [28,29,30,31]. A possible explanation could be found in the increased renal permeability to zonulin in HF patients, characterized by renal impairment. In particular, in the study of Dschietzig and co-workers [29], conducted in a group of failing patients, the authors obtained similar results with a direct relationship between e-GFR and zonulin, inferring that low levels of this protein linearly correlated with the degree of renal impairment, resulting from increased urinary elimination of zonulin. Interestingly and clinically relevant, Hasslacher and coworkers found a significant positive correlation between zonulin and e-GFR and an inverse relationship between zonulin and proteinuria in a population of longstanding patients with type 2 diabetes mellitus, postulating that these findings can be attributable to a an increased renal elimination of zonulin [31]. Therefore, present data, together with those previously published, may help to affirm that in patients with kidney damage, despite the presence of a systemic inflammatory status, zonulin and, consequently, LPS (EAA) levels are not increased, as consequence of an increased zonulin renal permeability. This hypothesis is further confirmed by the fact that both hs-CRP and fibrinogen are inversely correlated with zonulin, LAL and EAA in the whole HF population; these data have a biological plausibility because HF patients have an increased inflammatory burden and a worse renal function compared to controls, explaining the lower values of gut permeability markers despite an increased inflammatory burden. Of note, we also found a direct and significant relationship between SBP and gut permeability biomarkers in the whole HF population, probably as an expression of a better renal perfusion and consequent reduced urinary zonulin elimination. 

## 5. Conclusions

In conclusion, the results of this study, conducted in a well characterized population of failing patients, help to extend current knowledge about the role of gut microbiota in the pathogenesis of the immune-mediated inflammation characterizing this setting of patients, leading to the possibility of considering HF as an inflammatory disease developing as the host response to a PAMP. In addition, our results, together with those obtained by other authors, should encourage a re-evaluation of the role of serum zonulin levels in patients with renal impairment.

Our study has some limitations. First, due to the small sample size, our findings need to be confirmed in wider studies. Second, it is important to remember that both EAA and LAL are validated test for intensive care unit patients to predict the risk of sepsis, and to detect bacterial LPS in parenteral drugs, respectively; thus, their use in other clinical conditions is not validated, and the clinical significance of the results obtained in different population has not been demonstrated. Further studies conducted by directly testing dosing LPS levels in patients’ serum are needed.

Additional investigations in wider populations are required to confirm our hypothesis, to better characterize and treat HF patients, aiming to delay the progression toward end-stage cardiac disease.

## Figures and Tables

**Table 1:** Anthropometric, biochemical, hemodynamic and echocardiographic characteristics of the study population divided into control group and failing patients, and HF-rEF and HF-pEF.

	Controls (n = 20)	HF (n = 80)	*p*	HF-rEF (n = 40)	HF-pEF (n = 40)	*p*
**Gender**, m/f	13/7	47/33	0.609	31/9	16/24	0.0007
**Age**, yrs	54.8 ± 4.9	64.5 ± 5.3	0.0001	64.2 ± 5.9	64.9 ± 4.6	0.279
**SBP**, mmHg	116.7 ± 8.1	124.9 ± 10.6	0.0001	119.5 ± 10.3	130.3 ± 7.8	0.0001
**DBP**, mmHg	75.5 ± 7.3	74.8 ± 6.4	0.336	72.2 ± 6.2	77.5 ± 5.6	0.0001
**Glucose**, mg/dL	88.4 ± 6.1	105.0 ± 9.8	0.0001	102.0 ± 10.5	107.9 ± 8.1	0.003
**Cholesterol**, mg/dL	195.0 ± 19.7	217.0 ± 14.9	0.0001	215.2 ± 16.2	218.8 ± 13.3	0.140
**LDL-Chol**, mg/dL	127.4 ± 20.1	142.6 ± 15.2	0.0001	142.3 ± 18.7	142.9 ± 10.9	0.431
**HDL-Chol**, mg/dL	55.1 ± 15.1	46.1 ± 7.5	0.0001	47.1 ± 6.5	45.2 ± 8.3	0.129
**Triglyceride**, mg/dL	89.4 ± 15.1	142.3 ± 20.3	0.0001	132.0 ± 21.5	152.7 ± 12.5	0.0001
**Creatinine**, mg/dL	0.81 ± 0.13	1.05 ± 0.14	0.0001	1.13 ± 0.13	0.97 ± 0.11	0.0001
**e-GFR**, mL/min × 1.73 m^2^	96.3 ± 8.9	67.5 ± 11.4	0.0001	64.8 ± 9.6	70.2 ± 12.6	0.017
**Uric acid**, mg/dL	3.9 ± 0.7	6.1 ± 0.7	0.0001	6.4 ± 0.8	5.9 ± 0.4	0.0001
**NT-proBNP**, pg/mL	62.6 ± 16.1	401.1 ± 312.7	0.0001	683.4 ± 177.8	118.9 ± 55.9	0.0001
**EAA**	0.47 ± 0.18	0.25 ± 0.07	0.0001	0.22 ± 0.07	0.27 ± 0.07	0.001
**LAL**, EU/mL	6.15 ± 2.33	3.31 ± 0.95	0.0001	3.02 ± 0.80	3.60 ± 0.66	0.0001
**Zonulin**, ng/dL	21.9 ± 7.8	2.61 ± 0.46	0.0001	2.40 ± 0.40	2.82 ± 0.40	0.0001
**TLR2**, MFI	974 ± 228	1281 ± 310	0.0001	1409 ± 236	1147 ± 247	0.0001
**TLR4**, MFI	320 ± 152	469 ± 161	0.0001	539 ± 187	400 ± 74	0.0001
**WBCs**, 10^3^/uL	6.25 ± 0.91	7.78 ± 1.15	0.0001	8.78 ± 1.04	7.38 ± 1.12	0.0001
**Neutrophils**, %	60.8 ± 6.8	68.1 ± 5.6	0.0001	71.1 ± 4.9	64.9 ± 4.5	0.0001
**hs-CRP**, mg/L	1.93 ± 0.83	4.0 ± 1.6	0.0001	4.8 ± 1.3	3.2 ± 1.4	0.0001
**Fibrinogen**, mg/dL	137.0 ± 31.2	281.2 ± 68.2	0.0001	320.9 ± 66.1	241.5 ± 42.6	0.0001
***Echocardiographic parameters***						
**EF**, %	66.1 ± 4.8	45.6 ± 13.6	0.0001	32.9 ± 4.7	58.3 ± 4.6	0.0001
**i-LVM**, g/m^2^	93.4 ± 10.3	135.2 ± 13.7	0.0001	130.7 ± 9.6	139.6 ± 15.8	0.001
**ed-LVID**, mm	51.1 ± 2.4	59.3 ± 5.3	0.0001	63.7 ± 4.9	54.8 ± 5.6	0.0001
**es-LVID**, mm	35.5 ± 2.1	48.3 ± 8.1	0.0001	55.8 ± 3.0	40.9 ± 2.9	0.0001
**IVS**, mm	9.5 ± 0.9	10.7 ± 1.7	0.001	9.3 ± 0.9	12.1 ± 1.2	0.0001
**PW**, mm	9.6 ± 0.8	10.3 ± 1.7	0.039	9.0 ± 0.6	11.7 ± 1.3	0.0001

DBP = diastolic blood pressure; EAA = endotoxin activity assay; ed-LVID = end-diastolic left ventricular interior diameter; EF = ejection fraction; e-GFR = estimated glomerular filtration rate; es-LVID = end-systolic left ventricular interior diameter; HDL = high-density lipoprotein; hs-CRP = high sensitivity C-reactive protein; i-LVM = indexed-left ventricular mass; IVS = interventricular septum; LAL = limulus amebocyte lysate; LDL = low-density lipoprotein; NT-proBNP = N-terminal brain natriuretic peptide; PW = posterior wall; SBP = systolic blood pressure; TLR = Toll-like receptor; WBC = white blood cells.

**Table 2:** Correlational analysis between gut permeability markers and different covariates in the four study groups.

	Controls r/*p*	HF r/*p*	HF-rEF r/*p*	HF-pEF r/*p*
***Zonulin***				
Ejection fraction	−0.02/0.923	0.43/0.001	−0.09/0.591	0.06/0.702
NT-proBNP	0.04/0.868	−0.421/0.0001	−0.102/0.531	0.307/0.054
Age	0.16/0.512	−0.03/0.799	−0.02/0.896	−0.12/0.449
SBP	0.03/0.897	0.33/0.003	−0.13/0.410	0.44/0.005
hs-CRP	−0.09/0.714	−0.28/0.012	0.06/0.714	−0.17/0.300
Fibrinogen	0.19/0.428	−0.30/0.007	−0.06/0.725	−0.01/0.936
TLR2	0.187/0.430	−0.051/0.652	0.192/0.235	0.248/0.124
TLR4	0.206/0.383	−0.036/0.751	0.232/0.150	0.239/0.137
***EAA***				
Ejection fraction	0.03/0.911	0.31/0.005	0.02/0.901	−0.14/0.389
NT-proBNP	−0.023/0.354	−0.311/0.005	−0.036/0.823	0.184/0.257
Age	0.16/0.513	−0.09/0.449	−0.13/0.435	−0.10/0.529
SBP	0.02/0.985	0.33/0.003	0.04/0.812	0.38/0.016
hs-CRP	−0.30/0.202	−0.28/0.013	−0.15/0.352	−0.09/0.567
Fibrinogen	0.08/0.751	−0.27/0.015	−0.16/0.338	0.01/0.945
TLR2	−0.149/0.532	0.226/0.044	0.465/0.002	0.493/0.001
TLR4	0.165/0.486	0.221/0.048	0.561/0.0001	0.330/0.038
***LAL***				
Ejection fraction	0.03/0.911	0.35/0.002	0.08/0.608	−0.15/0.367
NT-proBNP	−0.007/0.624	−0.327/0.003	−0.011/0.950	0.223/0.178
Age	0.16/0.513	−0.12/0.289	−0.17/0.286	−0.13/0.426
SBP	0.01/0.987	0.35/0.002	0.05/0.768	0.43/0.005
hs-CRP	−0.30/0.202	−0.32/0.004	−0.18/0.254	−0.14/0.376
Fibrinogen	0.08/0.751	−0.33/0.033	−0.18/0.253	−0.08/0.645
TLR2	0.198/0.121	0.127/0.263	0.355/0.024	0.411/0.008
TLR4	0.137/0.174	0.206/0.067	0.513/0.001	0.340/0.032

EAA = endotoxin activity assay; hs-CRP = high sensitivity C-reactive protein; LAL = limulus amebocyte lysate; NT-proBNP = N-terminal brain natriuretic peptide; TLR = Toll-like receptor; SBP = systolic blood pressure.

## Data Availability

Data are available upon specific request to the corresponding author.
